# Feasibility and assessment of outcome measures for yoga as self-care for minorities with arthritis: a pilot study

**DOI:** 10.1186/s40814-018-0248-x

**Published:** 2018-02-20

**Authors:** Kimberly R. Middleton, Michael M. Ward, Steffany Haaz Moonaz, Miriam Magaña López, Gladys Tataw-Ayuketah, Li Yang, Ana T. Acevedo, Zavera Brandon, Gwenyth R. Wallen

**Affiliations:** 1 National Institutes of Health, Clinical Center, 10 Center Drive, Room 2B01, Bethesda, MD 20892 USA; 2 National Institute of Arthritis and Musculoskeletal and Skin Diseases, 10 Center Drive, Bethesda, MD 20892 USA; 3 Maryland University of lntegrative Health, Laurel, MD USA; 4 University of California Berkeley School of Public Health, Berkeley, CA 94720 USA; 5 NIH/Rehabilitation Medicine Department, Bethesda, MD USA

**Keywords:** Yoga, Minority, Osteoarthritis, Rheumatoid arthritis, Feasibility study

## Abstract

**Background:**

While there is a growing interest in the therapeutic benefits of yoga, minority populations with arthritis tend to be under-represented in the research. Additionally, there is an absence of guidance in the literature regarding the use of multicultural teams and sociocultural health beliefs, when designing yoga studies for a racially diverse population with arthritis. This pilot study examined the feasibility of offering yoga as a self-care modality to an urban, bilingual, minority population with osteoarthritis (OA) or rheumatoid arthritis (RA), in the Washington, DC area.

**Methods:**

The primary objective of the study was to assess the feasibility of offering an 8-week, bilingual yoga intervention adapted for arthritis to a convenience sample of primarily Hispanic and Black/African-American adults. A racially diverse interdisciplinary research team was assembled to design a study to facilitate recruitment and retention. The second objective identified outcome measures to operationalize potential facilitators and barriers to self-care and self-efficacy. The third objective determined the feasibility of using computer-assisted self-interview (CASI) for data collection.

**Results:**

Enrolled participants (*n* = 30) were mostly female (93%), Spanish speaking (69%), and diagnosed with RA (88.5%). Feasibility was evaluated using practicality, acceptability, adaptation, and expansion of an arthritis-adapted yoga intervention, modified for this population. Recruitment (51%) and participation (60%) rates were similar to previous research and clinical experience with the study population. Of those enrolled, 18 started the intervention. For adherence, 12 out of 18 (67%) participants completed the intervention. All (100%), who completed the intervention, continued to practice yoga 3 months after completing the study. Using nonparametric tests, selected outcome measures showed a measurable change post-intervention suggesting appropriate use in future studies. An in-person computerized questionnaire was determined to be a feasible method of data collection.

**Conclusions:**

Findings from this pilot study confirm the feasibility of offering yoga to this racially/ethnically diverse population with arthritis. This article provides recruitment/retention rates, outcome measures with error rates, and data collection recommendations for a previously under-represented population. Suggestions include allocating resources for translation and using a multicultural design to facilitate recruitment and retention.

**Trial registration:**

ClinicalTrials.gov, NCT01617421

## Background

While there is a growing interest in the therapeutic benefits of yoga for the prevention and alleviation of symptoms related to disease [1], there is a need for more evaluation of its effectiveness, especially among minority populations. Lifetime yoga practitioners are more likely female, non-Hispanic white, and college educated [2]; however, Hispanic and non-Hispanic Blacks are increasingly using yoga in the USA [3]. This pilot study investigates the feasibility of offering a yoga intervention to an urban, bilingual, minority population receiving rheumatology care.

A feasibility study [4] was indicated due to the under-representation of minority and non-English speaking populations in yoga research, a lack of researched guidance into sociocultural health beliefs, and a lack of diverse research teams. The few studies in the literature which speak to the experiences of minorities who have access to yoga practices are not specifically related to arthritis diagnoses [5–7]. This pilot study was undertaken using a yoga intervention shown to have a positive effect on patients with arthritis [8], by a racially diverse interdisciplinary team of clinicians familiar with the study population [9], and researchers who studied the health behaviors and beliefs of this population [10, 11]. This paper discusses the findings obtained from implementing the intervention as proposed in the original protocol [9].

## Methods

### Participants and setting

Research participants were recruited from English- and Spanish-speaking patients receiving care from the National Institute of Arthritis and Musculoskeletal and Skin Disease (NIAMS) Community Health Clinic (CHC) rheumatology practice located in a racially diverse area within the Washington, DC metro region. All enrolled patients met the eligibility criteria: 18 years of age or older, enrolled in the NIAMS Natural History of Rheumatic Disease in Minority Communities study (ClinicalTrials.gov# NCT00024479), and a diagnosis of either osteoarthritis (OA) or rheumatoid arthritis (RA). Exclusion criteria included recent or planned joint surgery, use of assistive ambulatory devices, hyper-mobility, or unstable disease that could compromise participation in the study. Detailed eligibility criteria can be found in the protocol article [9]. Informed consent and baseline questionnaires were completed at the rheumatology clinic by trained bilingual interviewers.

### Intervention

This study used Hatha yoga (influenced by Integral, Iyengar, and Kripalu yoga) which includes postures (asanas), breathing techniques (pranayama), and meditation [9]. Biweekly, 60-min, bilingual yoga classes were offered for 8 weeks at a yoga studio in Washington, DC. Classes were kept small (3–10 participants) to allow for pose modifications as needed for each participant. The same instructor taught all classes with occasional substitution from the principal investigator (PI)/yoga instructor (approximately 2–3 times per cohort). The same 16-class manual was used from the previous randomized research study *Yoga for Arthritis*, conducted through Johns Hopkins University [8]. Participants were given instructions, bilingual manuals, and yoga equipment to encourage home practice. Participants were asked to keep journals to document the frequency and duration of home practice and their experience while on the study. After the last class, a yoga DVD and a list of local yoga studios were given to encourage continued practice.

### Objectives

The objectives of the pilot study were to determine: (1) the feasibility of adapting a previously studied arthritis-based yoga intervention for a bilingual ethnic minority patient group, (2) the appropriateness of specific physical function and patient-reported outcome measures, and (3) the feasibility of using computer-assisted self-interview (CASI) for data collection [9].

### Sample size and ethical aspects

Since there were no references for this specific population using a yoga intervention, an accrual ceiling of 20 participants (determined by those attending at least one class) was selected based upon discussions with NIAMS clinical staff, previous research with the population [11], and attrition rates from the previous yoga for arthritis study [8]. Approval to conduct the study was obtained through the National Institute of Diabetes and Digestive and Kidney Disease (NIDDK)/NIAMS intramural institutional review board (IRB).

### Feasibility criteria

This paper addresses the feasibility which includes the practicality, acceptability, adaptation, and expansion of implementing the pilot study protocol [4]. Practicality was evaluated to the extent that intervention could be implemented within the constrained resources, time, commitment, etc. Acceptability was evaluated primarily through participant journal entries and exit interviews comments, outlined in a previously published article [12]. Adaptation and expansion were evaluated using specific rates (recruitment, adherence, and completion), selected outcome measures, exit interviews, and field notes. Data collected about the intervention included class attendance, home practice, and the continuation of yoga practice. This study expands and adapts the Hopkins study, highlighting changes needed for a bilingual minority population and offering the intervention in a community location. The previous *Yoga for Arthritis* study was not specifically designed to address minority participation. While the study had a diverse participant pool, there were few Spanish speakers, and the strongest predictor of attrition was minority race [8]. The feasibility of using a CASI data collection method was based on the amount of assistance needed, interviewer comments, technical issues, and exit interview comments.

### Outcome measures

**Fig. 1:**
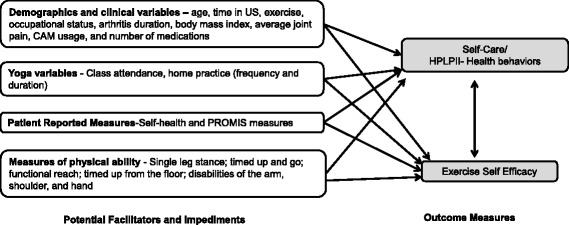
Conceptual model for potential relationships between facilitators/impediments, self-care, and exercise self-efficacy

Due to the lack of standard global measures to test the impact of the yoga intervention, a secondary objective was added to assess patient-reported outcome (PRO) measures and physical assessments (Table 1) across the two time points (baseline and final). Detailed information regarding the decision to use the selected measures were outlined in the original protocol article [9]. The two main outcomes, self-care and exercise self-efficacy (Fig. 1), were operationalized using the Health-Promoting Lifestyle Profile II (HPLP-II) [13] and the Chronic Disease Self-Efficacy–Exercise Regularly Scale [14]. Bandura’s social cognitive theory was used as a theoretical framework in determining paths of influence believed to be relevant for this study [15]. Social cognitive theory suggests that self-efficacy plays an important role in motivating behavior change. Self-efficacy is defined as the confidence in one’s ability to perform a task [15]. Self-efficacy beliefs shape the outcomes people expect their efforts to produce, also how obstacles and impediments are viewed. Those of high efficacy stay the course in the face of difficulties. Ways to influence self-efficacy include learning a new behavior, seeing people similar to oneself succeed, social persuasion, reducing negative emotional states, and correcting misinterpretations of physical ability [15].

**Table 1:** Patient-reported outcome and physical function measures

Measures	No. of items	Spanish translation	Reliability/validity tests in the literature
Self-Efficacy Exercise Regularly—Likert scale: 1 (not at all confident) to 10 (totally confident)	3	Y	(*n* = 478) mean 6.3 (SD 2.70). Internal consistency reliability 0.83. Test-retest reliability 0.86
Health Promoting Lifestyle Profile (HPLP II)—Likert-type scales (1—never to 4—routinely). Subscales: spiritual growth, interpersonal relations, nutrition, physical activity, health responsibility, and stress management.	52	Y	Alpha reliability coefficient for the total scale is .922. Alpha coefficients for the subscales range from .702 to .904.
Self-Rated Health—“Would you say your health in general is excellent, very good, good, fair, or poor?”	1	Y	(*n* = 51) Mean 3.29 (SD 0.91). Test-retest reliability- 0.92
PROMIS-29—8 domains include: anxiety, depression, fatigue, pain (interference and intensity), physical function, sleep disturbance, and satisfaction with social participation	29	Y	
Single Leg Stand (SLS)—determines if the patient can stand on one leg for 10 s.		N/A	Mean criterion-related validity was high (Pearson’s *r* = 0.84). Inter-observer reliability (ICC (2,1) = 0.81 (t1) and 0.82 (t2). Intra-observer reliability was on average ICC (2,1) = 0.88; Pearson’s r = 0.90
Functional Reach—measures the difference between a person’s arm length and maximal forward reach.		N/A	The Pearson correlation coefficient was .71 and the R2 using linear regression was .51; Test-retest reliability of the three postural control measures: intraclass correlation coefficient (ICC 1, 3) for center of pressure excursion (COPE) was .52, functional reach .81, and “yardstick” reach .92.
Timed Up and Go Test (TUG)—measures the time to stand up, walk 3 m, turn, walk back to the chair, and sit down.		N/A	Inter-rater reliability- ICC ranged from 0.99–0.992. Intra-session, test-retest reliability, ICC (2, 1) was 0.978
Timed Up from the Floor Test		N/A	The interrater reliability between various pairs of testers (*r* = .99)
The Disability of the Arm, Shoulder, and Hand (DASH)—measures generalized upper limb functional ability.		Y	DASH was found to correlate with other measures (*r* > 0.69) with a test-retest reliability of (ICC = 0.96)

The following additional measures were selected to operationalize potential facilitators or barriers for this population. Self-reported health [16] was selected as an attribute of overall well-being. The Patient-Reported Outcomes Measurement System (PROMIS) 29-profile [17] was selected to evaluate eight domains (Table 1) related to physical, mental, and social health while using fewer questions to reduce participant burden. Physical measures were selected and completed by the physiatrist, physical therapist, and occupational therapist on the study. Global physical measures (Table 1) were selected to evaluate domains of balance (single leg stance (SLS) [18], functional reach test [19]) and functional mobility (timed “Up and Go” test (TUG) [20]) that may be responsive to change with an exercise intervention. The timed floor transfer test [21] was used to evaluate strength, flexibility, function, and problem solving needed to transfer from standing to the floor and to return to standing. The Disabilities of the Arm, Shoulder, And Hand (DASH) [22] was selected to assess upper limb functional ability.

### Statistical methods

Descriptive statistics were used to report demographic and clinical measures. Spearman’s rho (for continuous variables) and Kruskal-Wallis test (for categorical variables) were used to test the relationships among demographic and clinical measures. The Wilcoxon signed-rank test was used to compare the difference from baseline to final time points for PROs and physical measures, for those completing the study (*n* = 12). “Change” variables were calculated using the difference (final minus baseline) across time. Linear mixed models were also used to evaluate change across the two time points using all available data for enrolled participants. Absolute values of correlation coefficients were used as estimates of effect sizes [23] using Cohen’s definitions for correlation coefficients: small (.10), medium (.30), and large (.50) [24]. Missing data were managed according to the measure recommendations. SPSS 21 statistical software was used for data analyses.

## Results

### Recruitment and participant flow

**Fig. 2:**
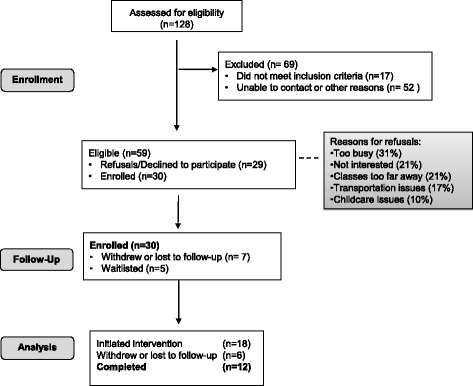
Flow Diagram

The principal investigator and bilingual research assistants attended the weekly outpatient clinic from 2012 through 2015. Patients were primarily referred by the NIAMS clinicians after completing their regular rheumatology appointment. Additional referrals from rehabilitation medicine clinicians were cleared by NIAMS clinicians prior to enrollment. The first participant was enrolled October 2012, and the first class started in March 2013. Initial classes were formed using only clinician referrals of frequently seen patients; however, after 18 months, direct referrals decreased. A list of less frequently seen, but potentially eligible referrals, was then provided for recruitment. Potential respondents were called directly, or “cold called,” using this list. This change in approach increased enrollment; however, without the initial discussion with a trusted clinician, refusals and ineligibles increased. After screening 128 patients, 51% of the 59 eligible patients were enrolled (Fig. 2). Reasons for refusals included the following: too busy, not interested, classes too far away, transportation issues, and childcare issues.

Once enrolled, participants completed a rehabilitation assessment and then were waitlisted until a cohort was formed. The yoga classes were offered as a group intervention, therefore scheduling was driven by the ability to create a cohort who could attend classes during the same times. Participants remained on a waiting list until a suitable cohort was formed. During the course of the study, six patients withdrew, including one for religious reasons [25]; additionally, seven patients were lost to follow-up. The start of the intervention was determined by attending the first yoga class; 60% of those enrolled met these criteria. Of the 18 participants who started the intervention, 12 completed (Fig. 2). Those who completed on average attended 10 out of 16 classes. Home practice averaged 2–3 days a week for approximately 20 min each day.

Selected demographic variables were evaluated for the difference between those enrolled and those who refused; crosstab chi-square values showed no significant different between the two groups. However, it was observed that of the two Hispanic interviewers on the study, one self-identified as “White” and the other as “Other.” Some participants appeared to choose a race category based on the interviewer’s interpretation, which may have been biased the selection of a race category. Additionally, there was no way to determine how this decision was made for refusal data taken from the medical record, as discussed further by Magaña López et al. [26].

### Study population

Data used to characterize the study population (Table 2) show enrolled participants (*n* = 30) were mostly female, Hispanic, with the predominant diagnosis of RA (90%). Most participants were foreign-born, with the largest percentage from El Salvador (36.7%). Acculturation was measured using the length of time in the USA (median 19.0 years) and a self-reported measure of English language proficiency which showed only 13.3% did not speak any English [27]. As opposed to previous yoga research with sedentary adults [8], as many as 83.3% participants reported engaging in some form of exercise, and half were employed. Using the Inventory of Complementary and Alternative Medicine Practices (ICAMP) [11], total counts of CAM usage showed a mean value of 6.3 (95% CI 4.9–7.6) per participant, with 23.3% reporting a “movement activity specifically for arthritis or joint symptoms.” Functional ability varied from those who walked twice a day for exercise, to those who needed a chair for yoga, and included some already receiving regular physical therapy for strength and flexibility. Group averages of painful joints using a response scale (0 = none thru 3 = severe) show approximately 50% reported no pain and as many as 6% reported severe pain at the time of enrollment (data not shown).

**Table 2:** Descriptive statistics (*n* = 30)

	Number	Percent
Gender		
Male	2	6.7
Female	28	93.3
Ethnicity		
Hispanic	21	70.0
Non-Hispanic or unknown	9	30.0
Race		
Black/African American	5	16.7
White	15	50.0
Other	5	16.7
Unknown	5	16.7
Place of origin		
North/East Africa, India, or Pakistan	4	13.3
Central America—Other countries	4	13.3
Central America—*El Salvador*	11	36.7
Mexico	3	10.0
South America	4	13.3
USA	4	13.3
English ability		
Very well	4	13.3
Well	10	33.3
Not well	12	40.0
Not at all	4	13.3
Health literacy		
SAHLSA (*n* = 20)		
Inadequate health literacy	9	45.0
Adequate health literacy	11	55.0
REALM-SF/grade equivalent (*n* = 10)		
(4–6)/seventh to eighth grade	2	20.0
(7)/high school	8	80.0
Diagnosis		
Osteoarthritis	3	10.0
Rheumatoid arthritis	27	90.0
Types of CAM**		
Health providers	4	13.3
Special diet	13	46.7
Vitamins or minerals	25	93.3
Herbs or supplements	13	43.3
Rubs or lotions	15	53.3
Other body treatments	6	20.0
Movement activity	7	23.3
Spiritual practice	19	6.0
BMI categories		
Underweight or normal (< 18.5–24.9)	6	20.0
Overweight (25.0–29.9)	12	40.0
Obese (> 30)	12	40.0
Exercise		
Sedentary	5	16.7
Occasional	7	23.3
Mild	16	53.3
Regular vigorous	2	6.7
Occupational status		
Employed—F/T	7	23.3
Employed—P/T	8	26.7
Unemployed, disabled, or retired	9	30.0
Homemaker	6	20.0
	Range	Median (IQR)
Age, years	32–69	49.5 (41.8–60.0)
Length of time in the USA, years	4–65	19.0 (10.0–27.5)
Arthritis duration, years	1–40	7.0 (4.0–15.0)
Body mass index	18.4–51.7	28.2 (26.1–33.4)
Yoga variables (*n* = 12)		
Total number of classes attended	4–16	10.5 (7.3–15.0)
Home practice frequency (days/week)	2–7	2.5 (2.3–4.8)
Home practice duration (min)	7–39	22.4 (14.8–31.4)

**Respondents could select more than one category; percent do not total to 100

### Evaluation of outcome measures

**Fig. 3:**
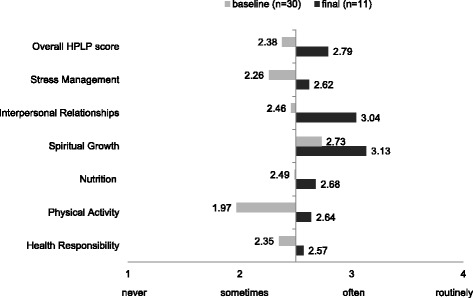
Distribution of Health-Promoting Lifestyle Profile II (HPLP-II) average values of overall score and subscales

Table 3 shows baseline values for patient-reported outcomes and physical measurements for those enrolled, along with baseline, final, and mean change values for those who completed the study. The average overall HPLP-II score showed a significant increase to 2.8 (2.5, 3.1) at the final time point. Using the corresponding labels (1 = never, 2 = sometimes, 3 = often, 4 = routinely), the average results for the six HPLP-II sub-scales (Fig. 3) show most baseline responses were below 2.5 at baseline, but increased closer to 3 after the intervention. Sixty-four percent (7 out of 11) of cases had an increased exercise self-efficacy score from baseline. Selected physical measures (Table 3) showed a significant change: SLS-left increased by 4.6 points and functional reach increased by 0.90 points indicating improved balance. DASH decreased by 14 points showing an improvement for this measure. Exploratory models using all available data showed an increase in self-efficacy (*p* = 0.008) and a decrease in self-rated health (*p* < 0.001) and DASH (*p* = 0.004) across time points.

**Table 3:** Physical measures and patient-reported outcome measures

Physical measures		Baseline enrolled (*n* = 26) Mean (95% CI)	Baseline (*n* = 12) Mean (95% CI)	Final (*n* = 12) Mean (95% CI)	Mean change * (12 pairs)
Single leg stance (s)	Left	15.0 (11.2,18.7)	12.2 (6.1,18.4)	16.9 (8.4,25.3)	− *4.61*
	Right	16.5 (12.4,20.6)	16.9 (10.6,23.2)	17.6 (8.9,26.4)	− 0.75
Functional reach (in.)		12.5 (11.7,13.3)	12.2 (10.9,13.5)	13.1 (11.7,14.6)	*− 0.90*
Timed up and go (s)		8.1 (7.4,8.8)	8.5 (7.7,9.3)	8.1 (6.8,9.4)	0.40
Timed Up from the Floor (s)^a^		7.7 (4.5,10.9)	9.6 (2.4,16.7)	6.0 (3.3,8.7)	3.58
Disabilities of the Arm, Shoulder, and Hand**		34.8 (26.4,43.1)	33.1 (20.9,45.3)	18.9 (11.9,25.8)	*14.23*
Patient-reported outcome measures		Baseline enrolled (*n* = 30) Mean (95% CI)	Baseline (*n* = 11) Mean (95% CI)	Final (*n* = 11) Mean (95% CI)	Mean change (11 pairs)
Self-rated health		3.4 (3.0,3.7)	3.0 (2.3,3.7)	2.1 (1.6,2.6)	0.91
Self-efficacy exercise		5.9 (4.9,6.9)	6.1 (4.0,8.1)	7.5 (6.8,8.1)	− 1.42
Health-Promoting Lifestyle Profile II score		2.4 (2.2,2.5)	2.3 (2.0,2.6)	2.8 (2.5,3.1)	*− 0.49*

*Wilcoxon signed-rank test. Italicized items are significant at *p* < 0.05

**For the DASH, lower scores equal less difficulty

^a^Final mean for Time Up from the Floor (*n* = 11), mean change calculated with 11 pairs

Average PROMIS-29 *T* scores were all within 1 SD of the US general population. *T* score distributions are standardized to compare to the average US population mean of 50 (SD—10 points) [17]. *T* score and standard error (SE) values (Table 4) were not directly calculated from the pilot sample but obtained from a Short Form Conversion Table based on raw scores from the pilot sample. While results for both time points are shown, the PROMIS-29 does not currently evaluate minimally clinically important differences and is not able to detect change related to the intervention. Only baseline measures were used in correlation evaluation.

**Table 4:** Patient-reported outcome measures—PROMIS Profile-29

PROMIS-29 profile (*T* scores)	Baseline enrolled (*n* = 30) Mean (SE)	Baseline (*n* = 11) Mean (SE)	Final (*n* = 11) Mean (SE)	Mean change * (11 pairs)
Physical function	45.3 (3.6)	46.0 (3.5)	47.4 (3.7)	− 1.37
Anxiety	54.5 (3.5)	51.7 (3.6)	49.1 (4.3)	2.68
Depression	53.5 (3.3)	49.7 (3.7)	50 (4.1)	− 0.30
Fatigue	50 (2.8)	48.4 (2.9)	49.7 (2.6)	− 1.32
Sleep disturbance	49.7 (3.7)	51.6 (3.8)	47.2 (3.6)	4.37
Satisfaction with social roles	48.3 (2.5)	47.4 (2.8)	50.6 (2.7)	− 3.22
Pain interference	56.9 (2.5)	55.5 (2.6)	49.7 (3.8)	*5.81*
Pain intensity (mean)	4.8	4.5	3.9	0.68

Pain intensity is a single item scored on a 0–10 scale; derived *T* score and SE values are not available

*Wilcoxon signed-rank test. Italicized item is significant at *p* < 0.05

#### Correlations

**Fig. 4:**
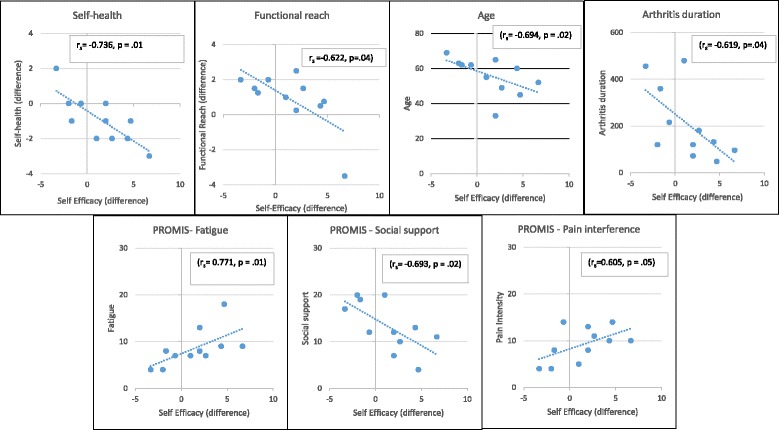
Scatterplot matrix and Spearman’s rho values for variables correlated with exercise self-efficacy

There were no significant correlations between any of the selected variables when compared to the change in HPLP-II. Significant correlations with the change in exercise self-efficacy are shown in (Fig. 4). Large effect sizes were found ranging from 0.604 (pain-interference) to 0.736 (self-health). There was an increased change in exercise self-efficacy for those with high reports of pain interference and fatigue at baseline. There was less change in exercise self-efficacy for older participants and those with longer disease duration. Increased exercise self-efficacy was correlated with improved self-rated health (from “fair” towards “very good” and “excellent”).

### Computer-assisted self-interview (CASI) data collection

All participants (*n* = 30) used the CASI instrument during the original enrollment process. On average, it took respondents 30 min with moderate assistance to complete the online questionnaire. Approximately, one third required 100% assistance; these participants typically also scored lower on the health literacy scales (Rapid Estimate of Adult Literacy in Medicine—Short Form (REALM-SF) and Short Assessment of Health Literacy for Spanish Adults (SAHLSA-50) [28]). Exit interviews indicated that respondents were “very comfortable/comfortable” (94%) using the CASI instrument and 100% would recommend its continued use.

### Exit interviews and 3-month follow-up

A semi-structured exit interview [9] was created to elicit participant feedback on the study design. For those participating in the intervention, exit interviews (*n* = 16) were completed in the yoga studio following the last class. An attempt was also made to complete the interview by phone for those who withdrew from the study after attending the first class. Most (94%) respondents indicated they were satisfied with the yoga classes and the studio and agreed yoga classes should be bilingual. Half agreed that they feel more comfortable taking classes from teachers with diverse racial/ethnic backgrounds; 85% agreed the yoga poses work well for people with arthritis; and 63% preferred taking classes with others who have arthritis. Follow-up results 3 months after the intervention found all (100%) of those who completed the study were still practicing yoga, for approximately 2–3 times per week.

#### Side effects/adverse events

There was one report of increased fatigue after starting yoga classes. Further investigation determined the participant was carrying yoga equipment and walking a great distance to save money. The solution was to obtain a reduced fare bus pass and use the studio equipment for class. There was one report of joint pain after practicing a modified reverse table pose that resolved by the next day. Participant was advised not to attempt the pose again.

## Discussion

This feasibility study was undertaken to evaluate the implementation of the intervention as proposed in the original protocol design. The discussion below summarizes findings related to feasibility including practicality, adaptation, and expansion to facilitate recruitment and retention, suggested future modifications, and a discussion of the study objectives.

### Adaptations for bilingual study population

The study team included bilingual researchers and clinicians from Central/South America, Mexico, and Puerto Rico. Two native “bicultural translators,” hired as research assistants, were involved from recruitment through data analysis. Their cultural familiarity assisted in establishing rapport, negotiating cultural understandings, and maintaining research integrity [29].

Several translation methods [30] were used. Team translation was used for Spanish journal entries and exit interview responses. Previously validated measures were most often translated using Spanish spoken in Mexico and Puerto Rico, instead of the Central American Spanish reflected within the study population. Professional translations were obtained for all remaining documents. When reviewed by native Spanish speakers, some changes were made to use less formal or non-anglicized wording. For example:

*English*: Touch and am touched by people I care about.

*Spanish*: Toco y soy tocado(a) por las personas que me importan.

While translated verbatim, the Spanish wording implies inappropriate physical touch. The phrase was translated as feeling “loved or connected to others” by the interpreter when administering the questionnaire. As documented in cross-cultural research [31], translating documents significantly increased cost. Using interpreters during data collection and yoga classes increased the amount of time needed to complete these phases of the study.

### Expansion for an urban, multicultural population

As discussed in the previous acceptability article [12], considerable time was spent designing a study with a “cultural infrastructure” to overcome potential barriers seen in the clinical setting and known from previous research with this population. Research team concerns with introducing yoga to this population included the lack of images of full-figured people or minorities doing yoga, the lack of familiarity with yoga, and few yoga studios directly within their communities. A daylong photoshoot was held to obtain culturally relevant images for the recruitment materials. Several of the study researchers and clinicians came to the yoga studio to assist with exit interviews which added to the connection between the yoga intervention and their clinical/medical care.

Based on previous experience with this study population, clinicians on the study expressed potential concerns about meeting required time points, which were found to be valid during implementation. There were several occurrences of rescheduling missed appointments due to inability to take time off from work, difficulty in obtaining childcare, or needing to provide family support. For some, frequent calls were initiated to inquire about missed classes and encourage continued participation.

Comparable to the previous *Yoga for Arthritis* study [9], attrition was highest prior to the first class (while waiting for a cohort to form). The majority of classes were held mid-day and early afternoon, with one weekend offering. Classes were not held during the winter months (December–March). Barriers most frequently identified by participants were the studio location was too far away and difficulty working class times into their work schedule. Some participants traveled as much as 1–2 h to get to the studio. Suggestions for future studies would be to offer travel vouchers, provide more evening/weekend classes, and on-site childcare to facilitate adherence while on the study. As stated earlier, clinician referral appeared to positively influence willingness to participate in the study. Calling from a list of potential participants increased access to a greater number of potential respondents but also increased refusals and ineligibles. It is suggested that both methods be employed in future studies.

### Appropriateness of selected measures and data collection methods

The values for self-care (HPLP) as well as physical measures of balance, functional reach, and upper body function showed a significant change after the intervention and should be considered in future studies. For this study, it was necessary to schedule separate appointments for questionnaire completion and physical assessment. It is possible that time needed schedule appointments may have been a barrier to participation or acceptability for some participants. This may be alleviated by combining assessments in future studies.

Exercise self-efficacy did not show a significant statistical difference. While not available when this study was created, the new Yoga Self-Efficacy Scale (YSES) [32] may provide improved results over the exercise self-efficacy scale used in this study. Age, fatigue, arthritis duration, and social support showed large effect sizes and significant statistical correlation with exercise self-efficacy in this population and should continue to be assessed in future studies. Some PROMIS measures, such as satisfaction with social support, did not correlate as expected. For example, the change in exercise self-efficacy decreased for those with increased satisfaction with social roles (Fig. 4). This runs counter to the importance of social support found in the previous health behavior study [33] and warrants further evaluation.

More precise measures of home practice are needed to determine a potential “yoga dosage.” Paper journals were used to decrease satisficing (which refers to a respondent providing a satisfactory answer instead of optimally answering a survey question that would require substantial cognitive effort) [34] and social desirability (inflating reports to be positively perceived) [35] of reported home practice. However, some respondents did not complete journals daily and may have reported weekly estimates of practice. Future research may consider the use of electronic diaries with an objective activity tracker (i.e., Fitbit®). Participants shared more about their experience, while in class with others, than in their journals as was expected. While collected in written field notes, future studies should consider audio-recordings.

Using the in-person web-based CASI questionnaires proved successful, especially after changing from a laptop to a tablet. The acquisition of a tablet (iPad) required less space in the busy clinic, and participants were more willing to use the touch screen. Additionally, a Wi-Fi hotspot was obtained due to unreliable internet connectivity. It is suggested that in-person CASI, via tablet administration, be used for future data collection.

### Generalizability

Due to the sample size limitations, results may not be generalizable; however, when appropriate, estimates are shown with confidence intervals which can be used to inform future sample size calculations [23]. Recommendations within this article offer suggestions for both expanding the current study design for a larger trial and for modifying other future yoga studies focusing on a bilingual minority population living with arthritis.

## Conclusions

Results of this pilot study determined that it is feasible to proceed to a larger study with suggested modifications. Recruitment and adherence rates were as expected based on previous experience with this population, and the previous *Yoga for Arthritis* study. Measures of balance, functional reach, upper body function, and health behavior showed change after completing the intervention. It is recommended that similar measures be included in future studies. Specific suggestions include using electronic diaries with an activity tracker, using the Yoga Self-Efficacy Scale, and providing identified options (i.e., childcare, transportation vouchers, and offering evening/weekend options) within the study design. In-person web-based CASI via tablet administration proved to be a feasible method for data collection. Three-month follow-up found all who completed the study were still practicing yoga. Overall suggestions include allocating additional time and resources for translation/interpretation for bilingual populations, as well as creating a multicultural interdisciplinary research team with previous clinical experience with the study population to facilitate recruitment and retention. This study provides recruitment and retention rates for a previously under-represented population. Results for selected measures are shown with error calculations for use in future sample size calculations.

## Abbreviations

CAM: Complementary and alternative medicine
CASI: Computer-assisted self-interview
CHC: Community Health Clinic
DASH: Disabilities of the Arm, Shoulder, and Hand
DC: District of Columbia
HPLP-II: Health-Promoting Lifestyle Profile II
ICAMP: Inventory of complementary and alternative medicine practices
IRB: Institutional review board
NIAMS: National Institute of Arthritis and Musculoskeletal and Skin Disease
NIDDK: National Institute of Diabetes and Digestive and Kidney Disease
NIH: National Institutes of Health
OA: Osteoarthritis
PI: Principal investigator
PROMIS: Patient-Reported Outcomes Measurement System
RA: Rheumatoid arthritis
REALM-SF: Rapid Estimate of Adult Literacy in Medicine—Short Form
SAHLSA-50: Short Assessment of Health Literacy for Spanish Adults—50
SLS: Single leg stance
SRH: Self-rated health
TUG: Timed “Up and Go” test

## References

[1] Jeter PE, Slutsky J, Singh N, Khalsa SB (2015). Yoga as a therapeutic intervention: a bibliometric analysis of published research studies from 1967 to 2013. Journal of alternative and complementary medicine (New York, NY).

[2] Cramer H, Ward L, Steel A, Lauche R, Dobos G, Zhang Y (2016). Prevalence, patterns, and predictors of yoga use: results of a U.S. nationally representative survey. Am J Prev Med.

[3] Clarke TC, Black LI, Stussman BJ, Barnes PM, Nahin RL. Trends in the use of complementary health approaches among adults: United States, 2002-2012. National health statistics reports. 2015(79):1–16. PMC4573565 25671660

[4] Bowen DJ, Kreuter M, Spring B, Cofta-Woerpel L, Linnan L, Weiner D, Bakken S, Kaplan CP, Squiers L, Fabrizio C (2009). How we design feasibility studies. Am J Prev Med.

[5] Wilson A, Marchesiello K, Khalsa SB (2008). Perceived benefits of Kripalu yoga classes in diverse and underserved populations. International Journal of Yoga Therapy.

[6] Saper RB, Boah AR, Keosaian J, Cerrada C, Weinberg J, Sherman KJ (2013). Comparing once- versus twice-weekly yoga classes for chronic low back pain in predominantly low income minorities: a randomized dosing trial. Evid Based Complement Alternat Med.

[7] Keosaian JE, Lemaster CM, Dresner D, Godersky ME, Paris R, Sherman KJ, Saper RB (2016). “We’re all in this together”: a qualitative study of predominantly low income minority participants in a yoga trial for chronic low back pain. Complementary therapies in medicine.

[8] Moonaz SH, Bingham CO, Wissow L, Bartlett SJ (2015). Yoga in sedentary adults with arthritis: effects of a randomized controlled pragmatic trial. J Rheumatol.

[9] Middleton KR, Ward MM, Haaz S, Velummylum S, Fike A, Acevedo AT, Tataw-Ayuketah G, Dietz L, Mittleman BB, Wallen GR (2013). A pilot study of yoga as self-care for arthritis in minority communities. Health Qual Life Outcomes.

[10] Wallen GR, Middleton KR, Miller-Davis C, Tataw-Ayuketah G, Todaro A, Rivera-Goba M, Mittleman BB (2012). Patients’ and community leaders’ perceptions regarding conducting health behavior research in a diverse, urban clinic specializing in rheumatic diseases. Prog Community Health Partnersh.

[11] Wallen GR, Middleton KR, Rivera-Goba MV, Mittleman BB (2011). Validating English- and Spanish-language patient-reported outcome measures in underserved patients with rheumatic disease. Arthritis Research & Therapy.

[12] Middleton KR, Magaña López M, Haaz Moonaz S, Tataw-Ayuketah G, Ward MM, Wallen GR (2017). A qualitative approach exploring the acceptability of yoga for minorities living with arthritis: ‘where are the people who look like me?. Complementary therapies in medicine.

[13] Walke S, Sechrist N, Karen R, Pender NJ. Health Promotion Model - Instruments to Measure Health Promoting Lifestyle: Health-Promoting Lifestyle Profile [HPLP II] (Adult Version). http://deepblue.lib.umich.edu/handle/2027.42/85349. Accessed 12 Feb 2018.

[14] Lorig K, Stewart A, Ritter P, González V, Laurent D, Lynch J. Chronic disease self-efficacy scales. http://www.selfmanagementresource.com/docs/pdfs/English_-_chronic_disease_self-efficacy_scales_32.pdf. Accessed 12 Feb 2018.

[15] Bandura A (2004). Health promotion by social cognitive means. Health Education and Behavior.

[16] Lorig K, Stewart A, Ritter P, González V, Laurent D, Lynch J. Self-rated health. http://www.selfmanagementresource.com/docs/pdfs/generalhealth.pdf. Accessed 12 Feb 2018.

[17] PROMIS Adult Profile Instruments [https://www.assessmentcenter.net/documents/PROMIS%20Profile%20Scoring%20Manual.pdf]. Accessed 12 Feb 2018.

[18] Haupstein T, Goldie P (2000). Visual judgements of steadiness in one-legged stance: reliability and validity. Physiother Res Int.

[19] Whitney SL, Poole JL, Cass SP (1998). A review of balance instruments for older adults. The American Journal of Occupational Therapy.

[20] Podsiadlo D, Richardson S (1991). The timed “up and go”: a test of basic functional mobility for frail elderly persons. J Am Geriatr Soc.

[21] Wang CY, Olson SL, Protas EJ (2005). Physical-performance tests to evaluate mobility disability in community-dwelling elders. J Aging Phys Act.

[22] Beaton DE, Katz JN, Fossel AH, Wright JG, Tarasuk V, Bombardier C (2001). Measuring the whole or the parts? Validity, reliability, and responsiveness of the disabilities of the arm, shoulder and hand outcome measure in different regions of the upper extremity. J Hand Ther.

[23] Maher JM, Markey JC, Ebert-May D (2013). The other half of the story: effect size analysis in quantitative research. CBE Life Sciences Education.

[24] Cohen J (1992). A power primer. Psychol Bull.

[25] Middleton KR, Andrade R, Moonaz SH, Muhammad C, Wallen GR (2015). Yoga research and spirituality: a case study discussion. Int J Yoga Therap.

[26] Magana Lopez M, Bevans M, Wehrlen L, Yang L, Wallen GR. Discrepancies in race and ethnicity documentation: a potential barrier in identifying racial and ethnic disparities. J Racial Ethn Health Disparities. 2016; 10.1007/s40615-016-0283-3 PMC5342943 27631381

[27] Language Use, About this Topic .http://www.census.gov/topics/population/language-use/about.html. Accessed 12 Feb 2018.

[28] Health Literacy Measurement Tools (Revised) [http://www.ahrq.gov/professionals/quality-patient-safety/quality-resources/tools/literacy/index.html]. Accessed 12 Feb 2018.

[29] Berman RC, Tyyska V (2011). A critical reflection on the use of translators/interpreters in a qualitative cross-language research project. Int J Qual Methods.

[30] Harkness JA, Braun M, Edwards B, Johnson TP, Lyberg LE, Mohler PP, Pennell BE, Smith TW. Survey methods in multinational, multiregional, and multicultural contexts. Hoboken: Wiley; 2010. http://www.worldcat.org/title/survey-methods-in-multinational-multiregional-and-multicultural-contexts/oclc/632157457. Accessed 12 Feb 2018.

[31] Liamputtong P. Doing cross-cultural research : ethical and methodological perspectives, 1 edn. Dordrecht: Springer; 2008. http://www.worldcat.org/title/doing-cross-cultural-research-ethical-and-methodological-perspectives/oclc/288440307. Accessed 12 Feb 2018.

[32] Birdee GS, Sohl SJ, Wallston K (2016). Development and psychometric properties of the Yoga Self-Efficacy Scale (YSES). BMC Complement Altern Med.

[33] Brooks AT, Andrade RE, Middleton KR, Wallen GR (2014). Social support: a key variable for health promotion and chronic disease management in Hispanic patients with rheumatic diseases. Clinical medicine insights Arthritis and musculoskeletal disorders.

[34] Krosnick JA (1991). Response strategies for coping with the cognitive demands of attitude measures in surveys. Appl Cogn Psychol.

[35] Nolte S, Elsworth GR, Osborne RH (2013). Absence of social desirability bias in the evaluation of chronic disease self-management interventions. Health Qual Life Outcomes.

